# Hypoxia down-regulates expression of secretory leukocyte protease inhibitor in bronchial epithelial cells via TGF-β1

**DOI:** 10.1186/s12890-015-0016-0

**Published:** 2015-03-07

**Authors:** Lisa I Påhlman, Annika Jögi, Magnus Gram, Michiko Mori, Arne Egesten

**Affiliations:** Department of Clinical Sciences Lund, Division of Infection Medicine, Lund University, Lund, Sweden; Lund University Cancer Center at Medicon Village, Translational Cancer Research, Department of Laboratory Medicine Lund, Lund University, Lund, Sweden; Department of Experimental Medical Sciences, Airway Inflammation and Immunology, Lund University, Lund, Sweden; Department of Clinical Sciences Lund, Respiratory Medicine and Allergology, Lund University, Lund, Sweden

**Keywords:** SLPI, Hypoxia, Airways, Pathogenesis, COPD, CF

## Abstract

**Background:**

Secretory leukocyte protease inhibitor (SLPI) is a protein with anti-protease and antimicrobial properties that is constitutively secreted from the airway epithelium. The importance of maintaining a balance between proteases and anti-proteases, and robust innate defence mechanisms in the airways, is exemplified by inflammatory lung conditions such as chronic obstructive pulmonary disease (COPD) and cystic fibrosis (CF). Both conditions present with a high protease burden in the airways which leads to tissue destruction. These patients also have an impaired innate immune system in the lungs with bacterial colonization and frequent airway infections. Moreover, both diseases are associated with airway hypoxia due to inflammation and mucus plugs. The aim of the present study was to investigate the role of hypoxia on SLPI production from the airway epithelium.

**Methods:**

Primary human bronchial epithelial cells were grown in sub-immersed cultures or as differentiated epithelium in air liquid interface cultures. Cells were incubated at 21% O_2_ (normoxia) or 1% O_2_ (hypoxia), and the release of SLPI was analysed with ELISA. RT-PCR was used to study the expression of SLPI and transforming growth factor β1 (TGF-β1).

**Results:**

Hypoxia decreased the constitutive production of SLPI by bronchial epithelial cells. The multifunctional cytokine TGF-β1, which is known to affect SLPI expression, showed increased expression in hypoxic bronchial epithelial cells. When bronchial epithelial cells were exposed to exogenous TGF-β1 during normoxia, the SLPI production was down-regulated. Addition of TGF-β1-neutralizing antibodies partially restored SLPI production during hypoxia, showing that TGF-β1 is an important regulator of SLPI during hypoxic conditions.

**Conclusions:**

The mechanism described here adds to our knowledge of the pathogenesis of severe pulmonary diseases associated with hypoxia, e.g. COPD and CF. The hypoxic down-regulation of SLPI may help explain the protease/anti-protease imbalance associated with these conditions and vulnerability to airway infections. Furthermore, it provides an interesting target for the treatment and prevention of exacerbation in these patients.

**Electronic supplementary material:**

The online version of this article (doi:10.1186/s12890-015-0016-0) contains supplementary material, which is available to authorized users.

## Background

Secretory leukocyte protease inhibitor (SLPI) is a small protein of 11.7 kDa that is secreted by mucosal linings, including the airway epithelium [1]. As the name implies, SLPI has anti-protease activity and plays an important role in neutralizing enzymes such as neutrophil elastase in order to prevent excessive tissue damage during inflammation [2]. Moreover, the protein also possesses antimicrobial activity against gram-positive and gram-negative bacteria, mycobacteria and fungi [3-6], although the mechanisms behind its antimicrobial properties are not fully understood.

Chronic obstructive pulmonary disease (COPD) and cystic fibrosis (CF) are two conditions affecting the lungs. Although the pathophysiology differs between the two diseases, they have several clinical manifestations in common. COPD is typically caused by long-term exposure to cigarette smoke, resulting in deterioration of lung function. In severe cases, these patients usually require long-term oxygen treatment due to hypoxaemia [7]. CF, on the other hand, is caused by a defect in the *CF transmembrane conductance regulator* gene, resulting in impaired chloride transport and excess mucus production [8]. Inflammation and infection are hallmarks of both diseases, and also major causes of the deterioration of respiratory function. The pathogenesis of both conditions is believed to involve a protease-antiprotease imbalance, where the protease activity from inflammatory cells is not adequately counter-acted by anti-proteases [9,10]. Moreover, bacteria often colonize the lungs of these patients, and they frequently suffer from airway infections and exacerbation that have profound effects on morbidity and mortality [11-14]. Given that SLPI has both antimicrobial and antiprotease properties, regulation of the protein may have important implications for disease progression.

Transforming growth factor β1 (TGF-β1) is a pleiotropic, multifunctional growth factor with fibrogenic and immunomodulatory properties. The protein has been implicated as an important regulator in the pathogenesis of inflammatory pulmonary conditions such as COPD, and an increase in TGF-β1 signalling has been seen in the lung in several studies on COPD (for a review, see [15]). Interestingly, TGF-β1 has also been shown to regulate SLPI expression [16].

In healthy tissues, the oxygen tension is normally between 2.5 and 9%, corresponding to 20–70 mmHg oxygen. During infections and inflammatory processes, oxygen is consumed by inflammatory cells at the infectious site, resulting in a hypoxic microenvironment with oxygen levels below 1% (<8 mmHg) [17,18]. COPD and CF patients have hypoxic areas in their lungs due to mucus plugs in the bronchi, chronic inflammation and tissue remodelling, resulting in shunting of blood from poorly to well ventilated areas. It is known that hypoxia increases the inflammatory function of inflammatory cells [18], but very little is known about the effects of hypoxia on the production of anti-proteases such as SLPI in the airway epithelium.

The present study was performed to investigate the impact of hypoxia on the secretion of SLPI by the respiratory epithelium. The results show that SLPI is down-regulated in bronchial epithelial cells in response to hypoxia, and that this is mediated via a TGF-β1-dependent mechanism.

## Methods

### Chemicals and reagents

Recombinant human TGF-β1 was purchased from Millipore (Darmstadt, Germany). Neutralizing monoclonal antibody against TGF-β1 and corresponding isotype IgG were obtained from R&D Systems (Minneapolis, MN, USA).

### Cells and culturing conditions

Primary human bronchial epithelial cells from non-smokers (3H Biomedical, Uppsala, Sweden) were grown in BEGM medium supplemented with BEGM bullet kit (Lonza, Basel, Switzerland) in poly-l-lysine-coated flasks (Sigma, St. Louis, MO, USA). The cells were seeded in 24-well plates (Sigma) coated with PurCol® (Advanced BioMatrix, San Diego, CA, USA) (1:100 v/v in H_2_O). Cells were grown until they were confluent and then incubated for 72 h with or without different stimuli under normoxia (21% O_2_, 5% CO_2_) or hypoxia (1% O_2_, 5% CO_2_). Hypoxic incubation was performed in a C-Chamber (BioSpherix, Lacona, NY, USA). After 48 h of incubation, the medium was replaced with fresh medium including stimuli. This medium had been pre-incubated in the cell incubator under hypoxic or normoxic conditions. At the end of the incubation period, the cell medium was collected and stored at −80°C until analysis.

For the growth of air liquid interface (ALI) cell cultures, Transwell® inserts in a 12-well plate were coated with 300 μl PurCol (1:100 v/v in H_2_O) for 20 min. Primary human bronchial epithelial cells were thereafter seeded onto the inserts and maintained in BEGM medium in sub-immersed cultures. When confluence was reached, the apical medium was removed and the baso-lateral medium was replaced by ALI medium, consisting of one part BEGM medium supplemented with BEGM bullet kit and 3 mg/ml BSA (Sigma), and one part D-MEM (Invitrogen, Walthem, MA, USA) supplemented with 1 mM sodium pyruvate MEM, 2 mM L-glutamine and non-essential amino acids (all from Invitrogen). The cell medium was thereafter changed at least 5 times/week. After 3–4 weeks in ALI culture, the cells were incubated at 21% or 1% O_2_ as above. Hypoxic incubation of the ALI cultures was performed with a glove box, InvivO_2_ Hypoxia 400 workstation (Ruskinn Technology Ltd, Bridgend, UK), to allow work under hypoxic conditions. After 48 h of incubation, the baso-lateral medium was changed and the apical surfaces were rinsed with PBS to remove excess mucus and pre-formed peptides. Incubation was terminated after 72 h by rinsing the apical surfaces with 100 μl 10 mM Tris buffer with 5 mM glucose. The rinsing fluids were stored at −80°C until analysis.

### SLPI detection

SLPI was detected in cell medium supernatants and rinsing fluids with ELISA (R&D Systems) according to the manufacturer’s protocol. As the volumes of the medium and rinsing fluids were known, the total amounts of SLPI released apically and baso-laterally could be compared.

### RNA isolation and real-time PCR

Total RNA was isolated from primary human bronchial epithelial cells using the acid guanidinium phenol chloroform method and the RNeasy Mini Kit supplied by Qiagen (Hilden, Germany). The optical density ratio (OD 260 nm/OD 280 nm) of RNA was always greater than 1.95. Reverse transcription was performed according to the manufacturer’s instructions on 1 μg total RNA using an iScript^TM^ cDNA Synthesis Kit (Bio-Rad, Hercules, CA, USA). RT-PCR was then used to quantify TGF-β1 and SLPI mRNA expression with primer pairs from OriGene Technologies (Rockville, MD, USA). Data were normalized to succinate dehydrogenase complex subunit A. This was chosen as internal standard based on analyses of the expression of a panel of 7 genes commonly used for normalization under hypoxic conditions (data not shown). Expression was analysed using the iTaq™ Universal SYBR® Green Supermix (Bio-Rad). Amplification was performed at 55°C for 40 cycles in an iCycler Thermal Cycler (Bio-Rad) and data were analysed using iCycler iQ Optical System Software (Bio-Rad).

### Neutrophil elastase activity assay

Neutrophil elastase activity was analysed with the chromogenic substrate N-methoxysuccinyl-Ala-Ala-Pro-Val p-nitroanilide (Sigma). The baso-lateral cell medium from ALI-cultures was diluted 1:4 in HEPES buffer (0.1 M HEPES pH 7.5 + 0.5 M NaCl). Fresh cell medium was used as a control. Equal amounts of diluted cell medium and 0.05 U/ml neutrophil elastase in HEPES buffer (40 μl each) (Sigma, St. Louis, MO, USA) were mixed in a 96-well plate. Samples were incubated at room temperature for 20 min. To each well, 100 μl of 0.2 mM N-methoxysuccinyl-Ala-Ala-Pro-Val p-nitroanilide in HEPES buffer was added. The plate was incubated at room temperature, and the absorbance was determined at 405 nm at different time points.

### Statistical analysis

Statistical calculations were performed using Student’s *T*-test or one-way ANOVA. Differences were considered statistically significant at p < 0.05.

## Results

### SLPI expression in bronchial epithelial cells is reduced in response to hypoxia

**Figure 1:**
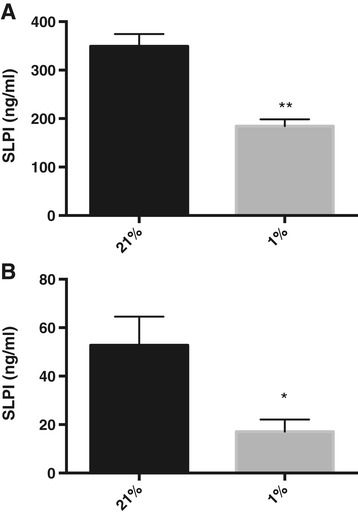
**Hypoxia reduces SLPI expression in differentiated bronchial epithelial cells.** SLPI concentrations in the cell medium **(A)** and rinsing fluid **(B)** from air liquid interface cultures incubated at 21% or 1% O_2_. The figure shows means and SEM for at least 3 independent experiments.

In order to study the effects of hypoxia on the secretion of SLPI in the airways, primary human bronchial epithelial cells were grown in ALI cultures. Under these conditions, cells differentiate to form a ciliated epithelium with mucus production similar to the *in vivo* airway epithelium. The differentiated cell cultures were incubated at 21% or 1% O_2_ for 72 h, after which the cell medium was collected, and the apical surfaces were rinsed to collect the periciliary liquid (PCL) including secreted proteins and peptides. Cells grown under hypoxic conditions were adherent and could not be morphologically distinguished from normoxic cells with light microscopy. The SLPI content in medium and rinsing fluids was analysed with ELISA. SLPI was mostly released into the baso-lateral medium (349 ± 25 ng/ml) compared to the rinsing fluid (53 ± 12 ng/ml) (Figure 1). However, if it is assumed that the depth of the PCL was 5 μm [19], the apical SLPI levels in the PCL would have been much higher at approximately 8 μg/ml, which is in good agreement with *in vivo* data [20]. SLPI concentrations were significantly reduced in both compartments in response to hypoxia (Figure 1).

**Figure 2:**
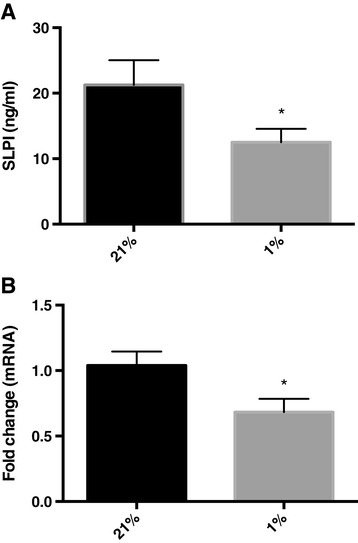
**SLPI expression in bronchial epithelial cells is reduced in response to hypoxia. A)** SLPI concentrations in cell medium from primary human bronchial epithelial cells grown under normal oxygenation (21% O_2_) or under hypoxic conditions (1% O_2_) (*n* = 5). **B)** SLPI mRNA levels from bronchial epithelial cells incubated at 21% or 1% O_2_. The values of fold change were calculated by normalizing to the data from the 21% O_2_ samples. The figure shows means and SEM of eight independent experiments.

We next investigated the effects of hypoxia on SLPI expression in non-differentiated primary bronchial epithelial cells grown in sub-immersed cultures. These cells were incubated at 21% or 1% O_2_, and SLPI concentrations in the cell medium were analysed after 72 h of incubation. SLPI levels were found to be reduced in cells grown under hypoxic conditions (Figure 2A), in line with the data from ALI-cultures. Cells were also harvested and analysed for mRNA expression using RT-PCR. The results showed that SLPI mRNA is down-regulated in response to hypoxia (Figure 2B), indicating that SLPI expression in a hypoxic environment is controlled at the transcriptional level, and is not a consequence of an overall reduction in metabolism.

Since the response to hypoxia was similar in both cell culturing systems, we continued to work with non-differentiated cells to facilitate the studies of underlying mechanisms.

### Hypoxia regulates SLPI expression via TGF-β1 signaling

**Figure 3:**
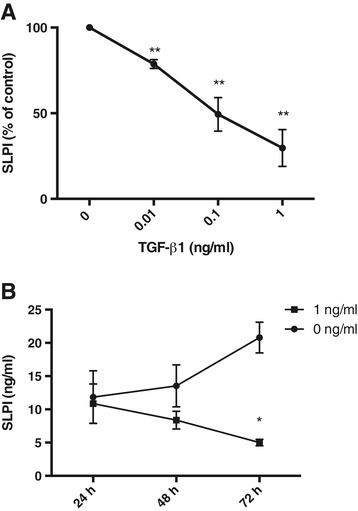
**SLPI expression is down regulated by TGF-β1.** SLPI concentrations in the cell medium from human bronchial epithelial cells stimulated with TGF-β1 **(A)**, or in the absence or presence of 1 ng/ml TGF-β1 **(B)**. The figure shows means and SEM of at least three independent experiments.

TGF-β1 is a growth factor that has been shown to down-regulate SLPI [16]. To confirm that SLPI can be regulated by TGF-β1 in the airways, bronchial epithelial cells were stimulated for 72 h with different concentrations of TGF-β1. SLPI concentrations in the cell medium were thereafter detected with ELISA. SLPI expression decreased with increasing TGF-β1 concentrations (Figure 3A), and the down-regulation of SLPI expression was more pronounced over time (Figure 3B).

**Figure 4:**
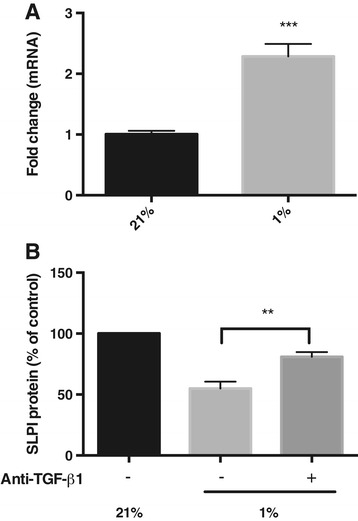
**Expression of TGF-β1 and SLPI in response to hypoxia. A)** Expression of TGF-β1 mRNA in bronchial epithelial cells cultured in 21% or 1% oxygen. The fold changes were calculated by normalizing to data from the 21% O_2_ samples (*n* = 4). **B)** SLPI concentrations in the cell medium from bronchial epithelial cells incubated at 21% or 1% O_2_ in the presence of a neutralizing antibody against TGF-β1 (50 μg/ml) or an isotype control antibody (*n* = 5).

In order to analyse the effect of hypoxia on TGF-β1 expression, RNA was isolated from cells grown in 21% and 1% oxygen, and the mRNA expression of TGF-β1 and SLPI was subsequently quantified using RT-PCR. TGF-β1 mRNA was significantly up-regulated in response to hypoxia (Figure 4A). Cells were then incubated in 21% or 1% O_2_, in the presence of a neutralizing antibody against TGF-β1. An isotype control antibody was used in parallel. Compared to the control antibody, SLPI expression in hypoxic cells was increased in response to anti-TGF-β1 (Figure 4B). Taken together, these results suggest that hypoxia down-regulates SLPI expression via up-regulation of TGF-β1 in bronchial epithelial cells.

### Medium from hypoxic cells is less potent in inhibiting neutrophil elastase activity

To investigate whether hypoxia affects the anti-protease property of the airway epithelium, baso-lateral cell medium from ALI cells stimulated with 21% or 1% oxygen was incubated with neutrophil elastase. Elastase activity was thereafter measured using a chromogenic assay. The results show a small but statistically significant difference between normoxic and hypoxic cell medium, where medium from normoxic cells inhibited elastase activity more efficiently (Additional file 1: Figure S1). These results indicate that hypoxia reduces the anti-protease potential of the airway epithelium.

## Discussion

**Figure 5:**
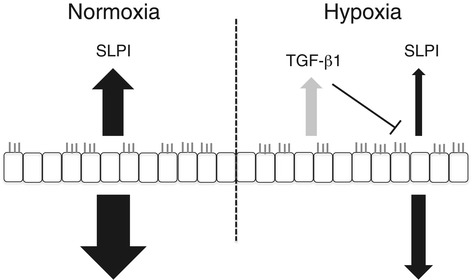
**Schematic model of hypoxia-driven SLPI expression.** During normal oxygenation, the bronchial epithelium constitutively secretes SLPI, both apically and baso-laterally. When the oxygen concentrations decrease, the epithelium increases its expression of TGF-β1, which in turn down-regulates SLPI production.

We have demonstrated a mechanism whereby SLPI is down-regulated in the airway epithelium in response to hypoxia (Figure 5). Under normal conditions, SLPI is constitutively expressed in epithelial cells. When oxygen is limited, hypoxia promotes up-regulation of TGF-β1, which in turn down-regulates SLPI.

When tissues are exposed to hypoxia, cells respond by up-regulating a number of genes, mainly through the hypoxia-inducible factor (HIF) pathways. HIFs are heterodimeric transcription factors that are rapidly degraded under conditions of normal oxygenation, but are stabilized and accumulate during hypoxia [21]. It has been reported that HIF-1α is up-regulated in the airways of patients with COPD and chronic bronchitis [22,23]. Although HIF-1α has been shown to regulate the TGF-β1 gene in mesenchymal stem cells [24], further studies are needed to investigate whether TGF-β1 and SLPI in the airways are regulated via HIF-1α or other hypoxia-driven pathways under hypoxic conditions. However, the increase in HIF-1α in the airways of COPD patients indicates that they have hypoxic regions in their lungs. Studies using direct oxygen measurements in the lungs of CF patients have demonstrated a hypoxic environment in the airway mucus, with mean oxygen levels as low as 2.5 mmHg [25]. SLPI concentrations in the airways of COPD and CF patients are reduced during bacterial exacerbation, and it has been postulated that the mechanism behind this observation involves proteolytic cleavage of the protein by neutrophil elastase [26-29]. Here, we propose a complementary mechanism whereby aggravated hypoxia during infection contributes to the reduction in SLPI concentration. Interestingly, it has also been reported that down-regulation of SLPI in COPD involves TGF-β1 signalling [30], which supports our model.

Conflicting data have been reported regarding the effects of hypoxia on TGF-β1 secretion. Boussat and co-workers found no change in TGF-β1 protein levels in the cell medium from pulmonary epithelial cells grown under hypoxic conditions [31], whereas others have reported an increase in TGF-β1 protein in response to hypoxia [24]. Our data show that neutralization of TGF-β1 with an antibody increases SLPI levels in hypoxic cells, suggesting that TGF-β1 concentrations are elevated under hypoxic conditions. Interestingly, Ambalavanan and co-workers showed that mice exposed to hypoxia exhibit an increased level of active TGF-β1 in their lungs, whereas total TGF-β1 concentrations were unchanged compared with controls [32]. This observation may explain the conflicting results on TGF-β1 levels in response to hypoxia.

SLPI was originally identified as a physiologically important inhibitor of neutrophil elastase and other proteases. Dysregulation of SLPI levels in the lungs of COPD and CF patients would therefore render them more vulnerable to damage caused by the inflammatory response. It is now known that the protein also has antimicrobial and immunomodulatory functions. SLPI has been shown to be effective against a number of gram-positive and gram-negative bacteria, including species common in the CF and COPD respiratory tract, such as *Pseudomonas aeruginosa* and *Staphylococcus aureus* [3,33]. Furthermore, SLPI can inhibit the inflammatory response by binding to lipopolysaccharide, thereby preventing macrophage activation [34]. This has been demonstrated *in vivo*, where SLPI-deficient mice challenged with lipopolysaccharide showed higher mortality and higher IL-6 expression than wildtype mice [35]. Moreover, clinical studies in which CF patients were given aerosolized recombinant SLPI have demonstrated that SLPI not only neutralizes neutrophil elastase activity, but also has an immunoregulatory effect, reducing IL-8 and neutrophil levels in the epithelial lining fluid [36,37]. This multifunctional role of SLPI makes it interesting from a therapeutic point of view, as altering this system could potentially reduce over-exaggerated proteolytic activity in inflammatory lung diseases such as COPD and CF, modulate the inflammatory response, and protect these patients from bacterial infections [38].

A limitation of the present study is that only epithelial cells were studied. Fibroblasts, endothelial cells and inflammatory cells were not studied, and their contributions to SLPI regulation in an *in vivo* setting are therefore unknown. Moreover, the experiments were carried out under non-inflammatory conditions. Further studies are thus needed to evaluate how hypoxia effects SLPI expression in the presence of normal airway microbiota, airway pathogens or other inflammatory stimuli.

## Conclusion

In conclusion, the present study demonstrates that SLPI is down-regulated in the bronchial epithelium during hypoxia. These findings may help explain the pathogenesis of inflammatory lung diseases, and provide a potential target for the treatment and prevention of exacerbation in these patients.

## Additional file

Additional file 1: Figure S1. Cell medium from hypoxic cells has reduced capacity to neutralize neutrophil elastase. Cell medium from air-liquid interface cultures exposed to normoxia or hypoxia were incubated with neutrophil elastase, and the elastase activity in the samples was thereafter analysed with a chromogenic assay. The figure shows neutrophil elastase (NE) activity in the presence of normoxic or hypoxic cell medium, compared to control medium (n = 6).

## Abbreviations

SLPI: Secretory leucocyte protease inhibitor
COPD: Chronic obstructive pulmonary disease
CF: Cystic fibrosis
TGF-β1: Transforming growth factor β1
ALI: Air liquid interface
PCL: Periciliary liquid
HIF-1α: Hypoxia-inducible factor-1α
