# Supplementary figures and images for: Hypoxia down-regulates expression of secretory leukocyte protease inhibitor in bronchial epithelial cells via TGF-β1
Source: BMC Pulm Med. 2015 Mar 7;15:19. doi: 10.1186/s12890-015-0016-0 (PMC4379733; doi:10.1186/s12890-015-0016-0)

# Suppl. Fig. 1

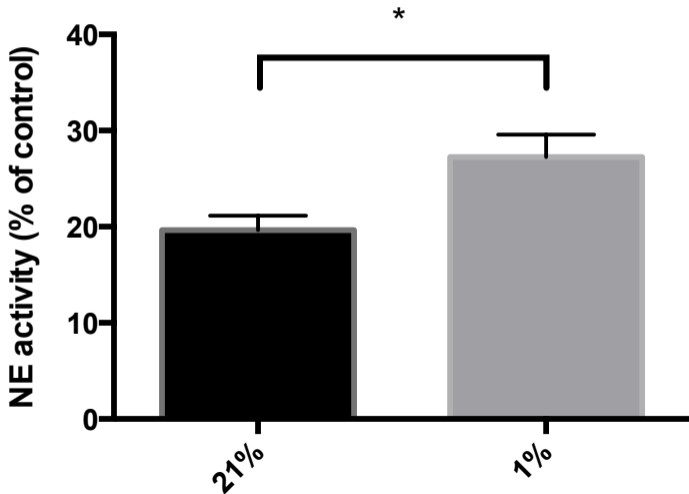

Supplement: Additional file 1: Figure S1. — Cell medium from hypoxic cells has reduced capacity to neutralize neutrophil elastase. Cell medium from air-liquid interface cultures exposed to normoxia or hypoxia were incubated with neutrophil elastase, and the elastase activity in the samples was thereafter analysed with a chromogenic assay. The figure shows neutrophil elastase (NE) activity in the presence of normoxic or hypoxic cell medium, compared to control medium (n = 6). [file 12890_2015_16_MOESM1_ESM.pdf]
